# Exploring Mitochondrial Heterogeneity and Evolutionary Dynamics in *Thelephora ganbajun* through Population Genomics

**DOI:** 10.3390/ijms25169013

**Published:** 2024-08-19

**Authors:** Haixia Li, Tong Liang, Yongju Liu, Pengfei Wang, Shaojuan Wang, Min Zhao, Ying Zhang

**Affiliations:** 1 State Key Laboratory for Conservation and Utilization of Bio-Resources in Yunnan, Yunnan University, Kunming 650032, China; lhx1005@mail.ynu.edu.cn (H.L.); liangtong@stu.ynu.edu.cn (T.L.); liuyongju@stu.ynu.edu.cn (Y.L.); 15288453604@sina.cn (P.W.); wsj20150915@163.com (S.W.); 2 School of Life Science, Yunnan University, Kunming 650032, China; 3 Institute of Highland Forest Science, Chinese Academy of Forestry, Kunming 650224, China

**Keywords:** mitogenome, wild edible fungi, persistent heteroplasmy, comparative population genomics

## Abstract

Limited exploration in fungal mitochondrial genetics has uncovered diverse inheritance modes. The mitochondrial genomes are inherited uniparentally in the majority of sexual eukaryotes, our discovery of persistent mitochondrial heterogeneity within the natural population of the basidiomycete fungus *Thelephora ganbajun* represents a significant advance in understanding mitochondrial inheritance and evolution in eukaryotes. Here, we present a comprehensive analysis by sequencing and assembling the complete mitogenomes of 40 samples exhibiting diverse *cox1* heterogeneity patterns from various geographical origins. Additionally, we identified heterogeneous variants in the *nad5* gene, which, similar to *cox1*, displayed variability across multiple copies. Notably, our study reveals a distinct prevalence of introns and homing endonucleases in these heterogeneous genes. Furthermore, we detected potential instances of horizontal gene transfer involving homing endonucleases. Population genomic analyses underscore regional variations in mitochondrial genome composition among natural samples exhibiting heterogeneity. Thus, polymorphisms in heterogeneous genes, introns, and homing endonucleases significantly influence mitochondrial structure, structural variation, and evolutionary dynamics in this species. This study contributes valuable insights into mitochondrial genome architecture, population dynamics, and the evolutionary implications of mitochondrial heterogeneity in sexual eukaryotes.

## 1. Introduction

Mitochondria are energy factories in eukaryotic cells and contain their genetic material. Their origin(s) and inheritance patterns play an important role in the evolution of eukaryotes [1]. Mitogenome size is much smaller compared with that of the nuclear genome; however, its structure is highly dynamic [2,3]. The size of mitochondrial DNA (mtDNA) ranges from 11 kb [4] to 531.2 kb [5], and the gene number ranges from 3 to 106 (excluding various mobile elements) [6]. This highly dynamic nature of mitochondria is primarily affected by the copy number of mitogenomes, gene structure and arrangement, gene duplication, dynamic introns, movable factors, and the recombination and length of intergenic regions [7]. This information is critical for deciphering the phylogenetic relationship and evolution of eukaryotes [8]. Recently, large-scale gene rearrangement, horizontal gene transfer, insertion, deletion, and inversion events have been detected among natural populations from fungal genera *Boletus*, *Agaricus*, and *Tricholoma* [9].

In fungi, mitochondrial DNA exists in a circular or linear form. There may be multiple copies in the same cell, and the mitochondrial function is closely related to the mtDNA copy number in cells [10]. Copy number polymorphisms in repetitive regions are an essential source of genetic diversity [11]. A recent genome-wide association study of 1011 natural isolates of *Saccharomyces cerevisiae* revealed that mitochondrial copy number variation is the largest genetic variation and has the greatest impact on phenotype [12]. Tang et al. hypothesized that the mtDNA copy number is inversely proportional to mtDNA size, cells thus maintain mtDNA quality rather than a constant number of genomes [13]. In addition, a few of the coding genes exhibit a multicopy pattern in mitochondria. For example, extra independent copies of the *rps3* gene have been discovered in several species of the genus *Coccidioides*. These additional copies may exist as distinct genes or may be introduced into the intronic region of the *rrnl* gene [14].

Mitochondrial introns are considered selfish genetic elements in most eukaryotes. They may invade homologous intron-free alleles, transpose to new sites, and horizontally enter the new genome to acquire beneficial functions and survive. The dynamics of introns have been reported in an increasing number of fungal species. For example, the comparative analysis of mitochondrial genomes of *Boletus*, *Tricholoma*, and *Auricularia* species revealed that different strains in the same species showed dynamic changes in introns (number and size). Frequent intron acquisition/loss events may further affect the evolutionary process of mitochondrial genomes and changes in their size [15]. Other studies have shown that intron insertion is a major reason for the relatively large mitochondrial genome of *Coniothyrium glycines* [16]. The maintenance of introns at specific insertion sites may be associated with adaptations of the host organism. For *Saccharomyces cerevisiae*, nuclear introns and the mitochondrial genome may contribute to tolerance in nutrient-deficient environments [17]. In addition, mitochondrial introns usually contain open reading frames (ORFs). The typical ORFs in introns are special DNA endonucleases that recognize and cut DNA-specific sites, which are known as HEs. HEs are mostly encoded by homing endonuclease genes (HEGs) in group I introns [18]. Based on the conserved amino acid motifs involved in the active sites of enzymes, there are four HE families: GIY-YIG, LAGLIDADG, His-Cys box, and HNH [19]. Among fungi, the most commonly observed HEs are LAGLIDADG(LD) and GIY-YIG(GIY), with the former being the most widely distributed [20]. HEs are rare cutting DNA endonucleases, unlike more common restriction enzymes, which target and cut DNA at frequently occurring sequences, they cut DNA at infrequent or unique sequences that are not commonly found throughout the genome, they have been used for genome editing and as molecular tools for targeted mutagenesis [21]. HEs also have applications in synthetic biology [22] as components of cloning vectors and cloning strategies [23].

The acquisition of introns may be obtained through homing pathways. Most introns contain intact or truncated ORFs associated with HEGs belonging to the LAGLIDADG and GIY-YIG families [24]. These HEGs are believed to be responsible for the intron movement [25]. HEGs have cutting and recognition activities and participate in the homing of introns. Intron homing refers to the movement of introns with HEGs to non-introns through various mechanisms, such as double-strand breaks, single-strand gaps, or homologous recombination [16]. Intron homing occurs at various levels and can be transferred between unrelated genes, alleles, or different chromosomes [16]. Intron homing results in the enlargement or acquisition of introns [25]. Because HEGs face negative selection pressure to remove selfish elements from the genome if they insert themselves into genes, their affiliation with introns enables them to remain in the genome [21]. The variation in HEGs is high and can generate genetic diversity not only through homing events and radical changes in the local mitochondrial genome structure where they insert but also through duplication [26]. Avoiding self-cleavage during conventional intron homing usually occurs by separating DNA binding sites and/or cleavage sites, which increases element mobility within the genome. Introns may also be acquired by horizontal gene transfer (HGT), in which introns spread horizontally between species or even kingdoms [27]. The fungal ancestor likely had introns and HEGs independently. Over time, HEGs and introns coevolved through recombination, transposition, and horizontal gene transfer. HEGs usually stay attached to the introns they invade, and mutations have primarily led to HEGs invading introns rather than becoming independent [16].

*T. ganbajun* is a prestigious edible mushroom grown exclusively in Yunnan Province, southwestern China. However, because of the restricted natural distribution of this endemic species, tight symbiotic associations with pine mycorrhiza, difficulties in artificial cultivation, and aggressive picking, the local population of *T. ganbajun* is showing signs of decline [28]. The market price of this mushroom can be as high as 200–280 USD per kilogram in wild mushroom markets in Yunnan Province during the rainy season from June to October. In a previous population genetic study of *T. ganbajun*, we found evidence of mitochondrial heteroplasmy in the *cox1* gene of basidiomycetes for the first time among natural populations of *T. ganbajun* in Yunnan [28]. This was characterized by the co-existence of two different introns in *cox1* with different insertion positions, lengths, copy numbers, and base composition. Furthermore, heteroplasmic single nucleotide polymorphisms (SNPs) were found in the *cox3* gene of minority *T. ganbajun* samples, suggesting that potential heteroplasmic loci may be present. Moreover, there is evidence of heteroplasmy and mitochondrial recombination repeatedly occurring in the natural populations of other wild edible fungi in Yunnan [29]. However, these findings are based on the analysis of a few mitochondrial gene loci by Sanger sequencing, and it is difficult to study the frequency of heterogeneity in the whole mitogenome of this species. The effect of heterogeneity on the structure and composition of the mitochondrial genome of the fungus and the extent to which it affects the genetic diversity of natural populations requires further study. In the present study, the complete mitogenomes of 40 *T. ganbajun* samples with various patterns of *cox1* heterogeneity and geographical origins, which were reported in our previous study, were sequenced and assembled. The structural variation and intron dynamics of the mitochondrial genome were compared. The variation in heterogeneous genes and the localization of homing enzymes were elaborated, which provides new evidence for the heterogeneous characteristics of mitochondrial genes in *T. ganbajun* and accesses the relationships between mitochondrial heterogeneity and genomic structure. Furthermore, SNPs in the complete mitochondrial genome were obtained to delineate the genetic structure and diversity of samples with heterogeneous mitochondrial genes and their relationships with geographical distribution.

## 2. Results

### 2.1. General Features of 40 Mitogenomes of T. ganbajun

The range in total length of each mitogenome of *T. ganbajun* was 64,130 bp to 68,859 bp and all were circular. The nucleotide composition of the mitogenomes was as follows: adenine: 37.10–37.32%, thymine: 36.65–37.05%, cytosine: 12.30–12.49%, and guanine: 13.37–13.74%. The GC content ranged from 25.69% to 26.23% with an A+T-rich feature. Each mitogenome had two ribosomal RNAs [one encoding the small subunit (*rrns*) and one for the large subunit (*rrnl*)]. The length of rnl in the different samples ranged from 3824 bp to 3887 bp; the largest had a length variation of 63 nucleotides. The length of all rns was 1802 bp, without any nucleotide length variation. The total number of transfer RNAs (tRNAs) was 34 to 38 per mitogenome, which differed slightly within the *T. ganbajun* species. Detailed information on all 40 mitochondrial genomes is listed in Table 1, while the geographic distributions of the samples are provided in Table 2.

### 2.2. Structure of Standard Protein-Coding Genes

**Figure 1:**
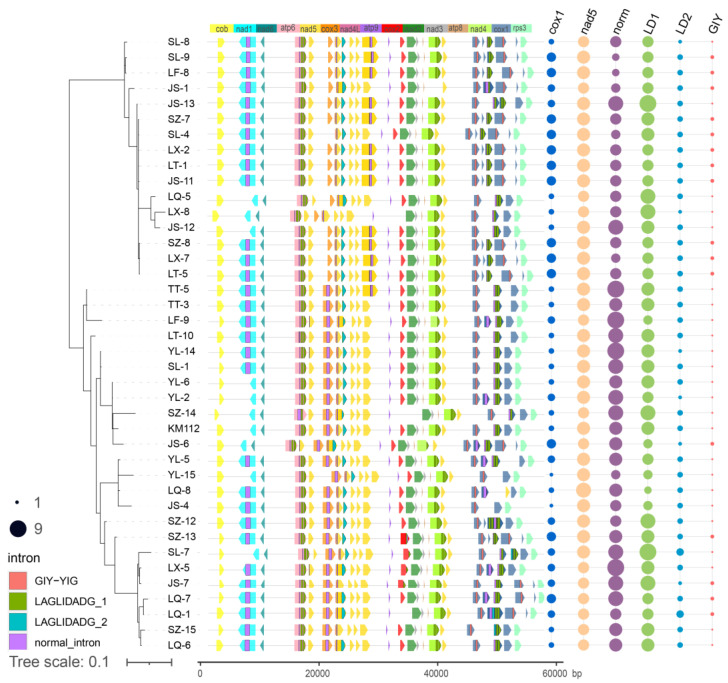
Structure of standard protein-encoding gene (PCG) and distribution of the numbers of introns and multicopy genes on a Bayesian inference (BI) tree constructed from 13 conserved PCGs and rps3. Note: Different colors indicate different genes, different lengths indicate the size of genes, and arrows indicate the direction of genes.

There were 21–25 standard PCGs per mitogenome of *T. ganbajun*. The structure and size of the standard PCGs for each sample are shown in Figure 1.. The structural ordering of the mitochondrial PCGs in all sequenced *T. ganbajun* samples was stable, except for LQ-8, in which one of the multicopy heterogeneous types (HTs) of *nad5* was inserted between two different HTs of *cox1* (Figure 1). This inserted copy was identical to that of the 3′ end of the fifth *nad5* copy of the same specimen. They had a highly similar sequence of 247 bp with a similarity of 87%. This was the only heterogenous fragment repeatability detected in the same gene of the same specimen. In addition, the 5′ end of the second heterogenous fragment of *nad5* in all specimens coincided with the 3′ end sequence of *nad4L*, which is highly similar to the middle part of *nad4L*. The two genes share a sequence fragment; however, this sequence is not completely similar, as shown in Figure 1. The fragments of the *nad4L* and *nad5* genes, represented by rose red and light orange, overlap. Using sample LQ-8 as an example, among the overlapping 267 bp bases, the similarity of the first 141 bp bases was 99%. There was only one gap difference, but no similarity in the last 126 bp. Certain PCGs were missing in a few samples. For example, JS-1 had no *nad4L* gene, whereas the *cox2* gene was absent in LQ-1, LX-8, and SZ-14, and there was no *cox3* gene in SL-4.

All PCGs started with the standard translation start codon ATG, except in *nad4L*, which started with ATT or ATA. The stop codon TAA was preferentially used, whereas TAG was also present. All anticodons in this study encoded 20 essential amino acids, with 159 arginine (TCG, CCG, TCT), 159 serine (CGA, TGA, GCT), and 129 isoleucine (GAT, AAT, TAT) as the most common.

### 2.3. Multicopy HTs Verification in cox1 and nad5

There were two genes, *cox1* and *nad5*, that had multicopy HTs. The total multicopy gene size ranged from 8428 to 12171 bp in each mitogenome and accounted for 37.45% to 50.56% of the total standard PCG size per mitogenome. The number of HTs in *nad5* was 6–8 per sample and the sizes of HTs in *nad5* gene in each sample were also different, with the largest 2558 bp and the smallest 600 bp, and the average HT size in *nad5* was 920 bp. The number of HTs in *cox1* in each sample was smaller, varying from 2 to 5. The largest *cox1* HT was 2630 bp, the smallest was 285 bp, and the average size was 1172 bp. Moreover, different copies of the two genes were heterogeneous, and different HTs had different copy numbers (Table S1).

**Figure 2:**
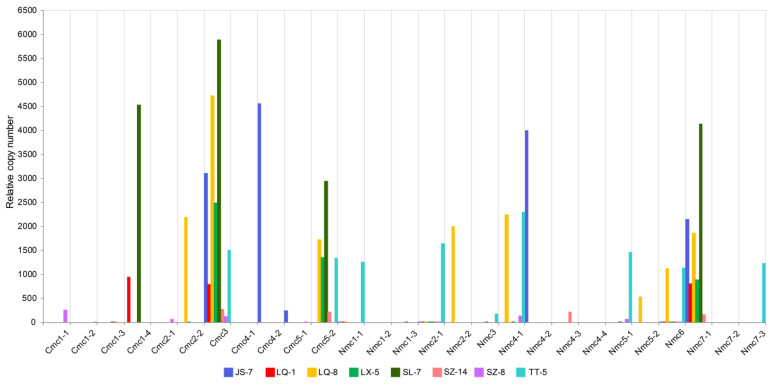
Relative copy number of mitochondrial multicopy genes. Notes: Names in the x-axis are the names of tested HTs by RT-PCR, fragments starting with C are the multicopy genes of *cox1*, and those starting with N are from *nad5*. The distributions of these multi-copy genes are listed in Table S2.

The Ct values of all HTs were normal. The amplification product was also single based on the melting peak, indicating that the results were available. The relative copy number value of each HT is shown in Figure 2 and Table S1. The relative copy number of different mitochondrial HTs for different strains varied greatly, with the smallest value of 0 and the largest reaching 5900.88. The relative copy number of HT #Nmc1-1 was 0 in SZ-14 and SZ-8, but it was 1266.07 in TT-5. As shown in Figure 2, each sample had many different HTs, which was consistent with a mixture of mtDNA haplotypes in single individuals, confirming the prevalence of heteroplasmy in the natural *T. ganbajun* populations. Although we failed to effectively amplify the target fragment from sample Nmc1-2 after several attempts, its existence cannot be denied. We hypothesized that the content of the gene fragment was low because, in these multicopy fragments, there are a large number of AT repeats and homologous sequences, thus non-specific amplification can occur.

### 2.4. Intron and HEs Dynamics of Mitogenomes

#### 2.4.1. Intron Distribution

**Figure 3:**
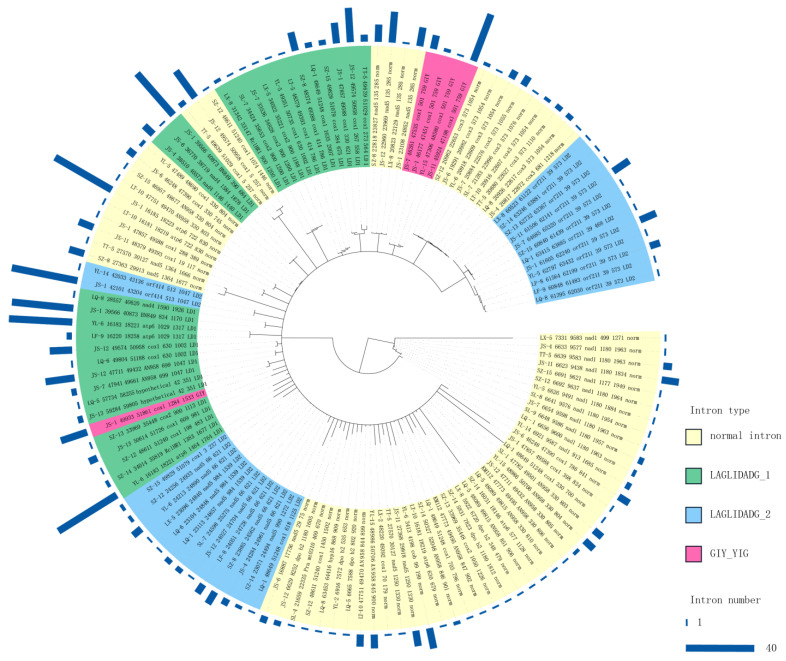
Maximum Likelihood (ML) phylogenetic analysis of all intron types (Nucleic acid sequences) from 40 *T. ganbajun* samples in this study. Notes: Due to the large number (707), the introns of the sequence duplicates are kept, and the excess sequences are deleted. The different colors of the inner ring indicate different types of introns, and the dark blue bar graph of the outer ring indicates the corresponding number of introns.

The distribution of introns in standard PCGs is uneven. A total of 707 introns were identified in 40 mitochondrial genomes, which were divided into four types based on whether they contained ORFs: normal introns (without ORF) and three types containing different nucleic acid HEs—the LD1 type (containing the LAGLIDADG_1 family), the LD2 type (containing the LAGLIDADG_2 family), and the GIY type (containing the GIY-YIG family). The most abundant introns identified were normal intron (271), followed by the LD1 type (264), the LD2 type (116), and the GIY type with only 56 (Figure 3). The number of normal introns per sample ranged from 4 to 9. The LD1 type ranged from 4 to 9, the LD2 type ranged from 2 to 4, and the GIY type ranged from 1 to 2. The number of HEs in each sample was at least 8 (3107 bp) and at most 14 (4949 bp).

We detected 76.8% (543/707) of the introns in the *cox1*, *nad5*, *atp6*, *nad4*, *cox3*, *nad1*, and *cox2* genes, and there were significant insertions and deletions of introns in the same host gene (e.g., *cox1*, *nad5*, *cox3*, *nad1*, and *cox2*) in different samples (Figure 1). Moreover, 23.2% of the introns were located in ORFs and other hypothetical protein-coding genes. Notably, among the 543 introns of the 15 standard PCGs, the number of LD1 introns was the largest at 235, followed by normal introns at 212, with the lowest number being 40 for the LD2 introns. In addition, introns present in two genes (*nad5* and *cox1*) with heterogeneity accounted for more than half of all 15 standard PCGs (288/543 = 0.53). LD2 was only present in *cox1* (3) and *nad5* (37), whereas all 56 GIYs were detected only in the *cox1* gene (Figure 3).

The distribution of HTs and the number of introns in different samples are shown in Figure 1. On the left is a Bayesian phylogenetic tree constructed based on 13 standard PCGs; the clustering of clades showed no geographical specificity. The figure on the right shows the position distribution and size of introns in the PGC gene structure. The intron distribution and gene structure variation were not significantly associated with the phylogenetic relationship. The number of identical introns within the same clade was inconsistent. The number of HTs was also inconsistent with the phylogenetic clustering, indicating that the distribution of introns and HTs was not correlated with the phylogenetic relationship of *T. ganbajun*.

#### 2.4.2. Intronic Polymorphism

Figure 3 shows the phylogenetic relationships of representative sequences of introns. When inferring the root by examining internal nodes, the intron type near the root of the phylogenetic tree was a normal intron with high sequence variation, and the number of introns represented by the dark blue bars in the outer circle was relatively small. The variation in the LD1 intron was relatively low, and the number of introns in the outer circle was relatively large. Introns of the same type were not all gathered in one clade but were cross-distributed among the clades of different types of introns. The same type of introns was occasionally clustered together, such as the two clusters of the GIY type, which were distributed in the same sample. However, most of the same intron types in the same host gene clustered in the same subcluster. For example, the LD2 type of introns clustered into different clades based on different host genes (orf211, orf414, nad5). In addition, the nucleotide diversity of GIY was 0.18, LD2 was 0.360, LD1 was 0.47, and the normal intron was 0.6. The genes with homing endonuclease were more conserved compared with those with normal introns.

The intron sequences inserted at the same site in the same host gene were generally similar but had some variations: (1) Inconsistent insertion sites within the same gene can lead to changes in length, with extra bases added at either the 3′ or 5′ end. For instance, at insertion site 722 of the *atp6* gene, the LT-10 intron had a 48 bp deletion at the 5′ end and a 48 bp addition at the 3′ end. (2) Different insertion sites in various host genes across samples may show partial sequence similarity. For example, normal introns with different insertion sites were partially conserved and clustered at the root of the phylogenetic tree, despite being found in ten different host genes (Figure 1 and Figure 3).

Although the difference in standard PCGs is primarily reflected in the size of the gene and the number of copies, the size of the PCGs was correlated with a gain or loss of introns (Figure 1). For example, the presence of normal introns usually made the gene larger, and the protein-coding genes without introns were smaller (e.g., *atp8*, *atp9*, *nad2*, *nad3*, *nad4l*, *nad6*, and *rps3*). In particular, the existence of introns resulted in changes in the size of HTs in the *cox1* and *nad5* genes, and the difference in the number and size of the HTs directly caused significant changes in these two genes. Figure 1 shows the distribution of heterogeneous genes and their introns. Two types of introns were found on the HT of *nad5*, which were normal introns and LD2, and the largest normal introns (371–476 bp) were present in the largest HT of *nad5*. Although the heterogenous *cox1* has four types of introns, among them, LD2 showed the smallest number. Moreover, the *cox1* gene contained all 56 GIYs identified in the present study and had two different length variations (259 and 250 bp). Each of the first HT of *cox1* in each sample had the former type, whereas only 16 samples had the latter type, which was located on the third or fourth *cox1* HT. Furthermore, the LD1 homing enzyme had four host genes. Among the three host genes (*atp6*, *nad4*, and *cox1*), two adjacent copies were distributed on the same gene, and there was only one copy on the *cox2* gene.

### 2.5. Correlation Analysis of the Main Characteristics of the Mitochondrial Genomes

**Figure 4:**
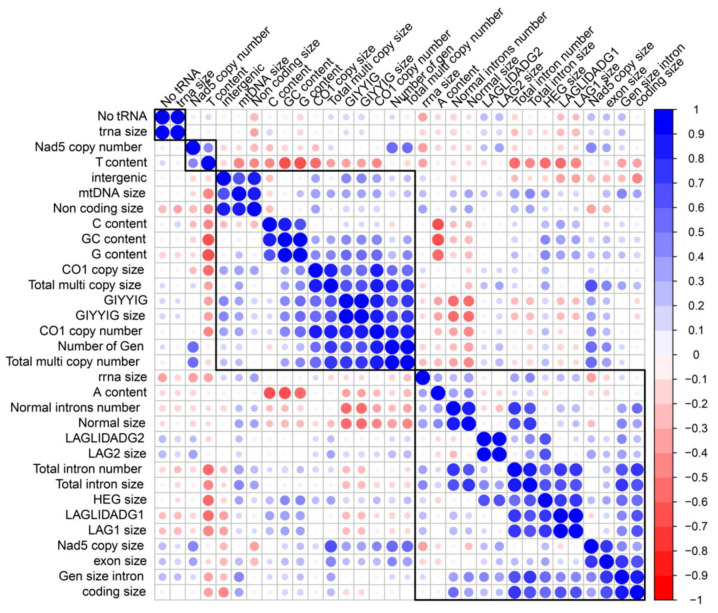
Correlation analysis of mitochondrial genome structure, composition, size of homing endonuclease genes and introns, and the existence of multicopy heterogeneous types in *cox1* and *nad5.* The sizes of the circle suggest R² value. Red and blue colors indicate the correlation. The blue color represents the trend of positive correlation, red represents a negative correlation.

The location, size, and number of HTs changed with the changes of introns. To further identify the origin of the widespread and persistent heteroplasmy in natural *T. ganbajun* populations, a correlation analysis of the main characteristics of the mitochondrial genomes was conducted. As shown in Figure 4, (1) the total length of the mitochondrial genome exhibited a significant correlation with the lengths of the non-coding region and the intergenic region; (2) the number of HTs for *cox1* was significantly correlated with the number and length of GIY; (3) the number and length of introns in the mitochondrial genome were significantly correlated with the size of the HEGs, particularly the number and size of LD1, because among the three homing endonucleases, LD1 had the highest number (4–9/sample) and the largest size (1222–2838 bp/sample); (4) the gain or loss and length of the introns affected the length of the protein-coding genes, and the existence of HEGs affected the composition of the mitochondrial genes.

### 2.6. Horizontal Gene Transfer of HEs

**Figure 5:**
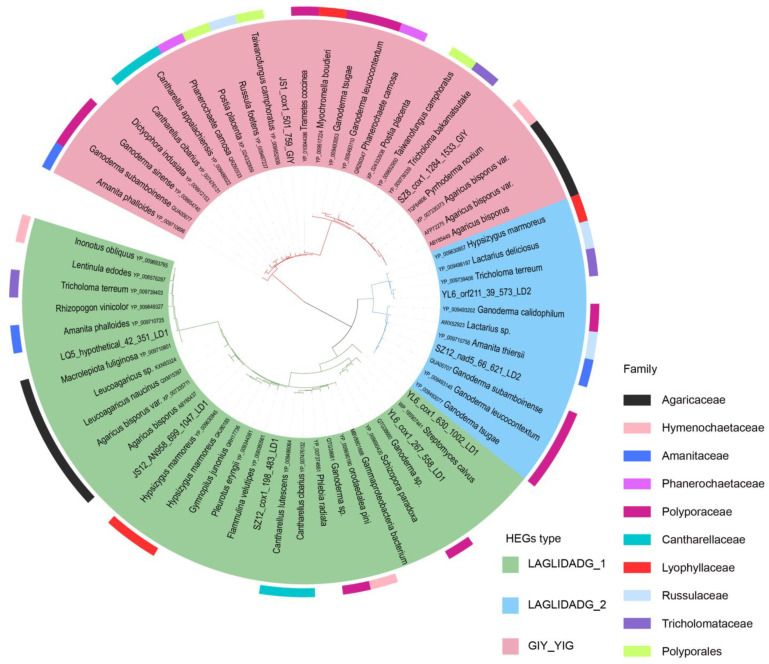
Phylogenetic analysis of horizontal gene transfer (HGT) of homing endonucleases. Note: Different colors in the inner circle indicate different types of homing endonucleases, and different colors in the outer circle indicate species in different families.

The sequences of the three types of HEs in the present study were compared with those from the NCBI, and the gene sequences with a coverage of >80% and a similarity of >70% were selected. Of these, 55 homologous sequences were identified, which belonged to ten homing enzymes (5 LD1, 3 LD2, 2 GIY), and were distributed in 29 genera and 39 species (Table S3). Most of the sequences (53) belonged to Agaricomycotina, one belonged to Actinobacteria (*Streptomyces calvus*), and one belonged to an unclassified Gammaproteobacterium in Proteobacteria. The similarity of the largest sequence compared with LD2 was 91%, and the coverage was 94%. The highest similarity with LD1 was 88%, and the coverage was 96%. The highest similarity with GIY was 86%, and the coverage was 96%. The homing enzyme sequence with high similarity to Actinomycetes (similarity 83%, coverage 95%) was LD1, and the sequence with high similarity to Proteobacteria (similarity 72%, coverage 84%) was also the LD1 homing enzyme. Bayesian phylogenetic analysis revealed that GIY, LD1, and LD2 are aggregated into three different major clades, each with different subclades. HEs of the same species may be aggregated into the same subclade or dispersed into different clades (Figure 5). In different clades, the distribution of these species has no certain rules. HEs with high homology are also found in species of different families.

### 2.7. Population Genetics

SNPs in the mitochondrial genomes of 40 *T. ganbajun* samples from four geographical groups (Honghe, Kunming, Qujing, and Chuxiong) were divided into four categories: all SNPs, intergenic SNPs, exon SNPs, and intron SNPs. Table 3 shows detailed information about these SNPs. There were 2463 SNPs, of which 1911 were parsimony informative, whereas the remaining 552 were singleton variable sites. The nucleotide polymorphism (Pi) of all SNPs was 0.23. Among the other three types of SNPs, the highest genetic diversity (Pi value, 0.263) occurred in the exon region. The Pi values of different populations in the same gene region were different. In the exon region, the Pi value of Honghe was the highest (0.282), and that of Qujing was the lowest (0.199). Moreover, the Pi values of the four different populations fluctuated in different gene regions. In the intergenic region, the largest Pi values occurred in Chuxiong, whereas in the exon and intron regions, the largest Pi values occurred in Honghe and Qujing, respectively (Table 3).

**Figure 6:**
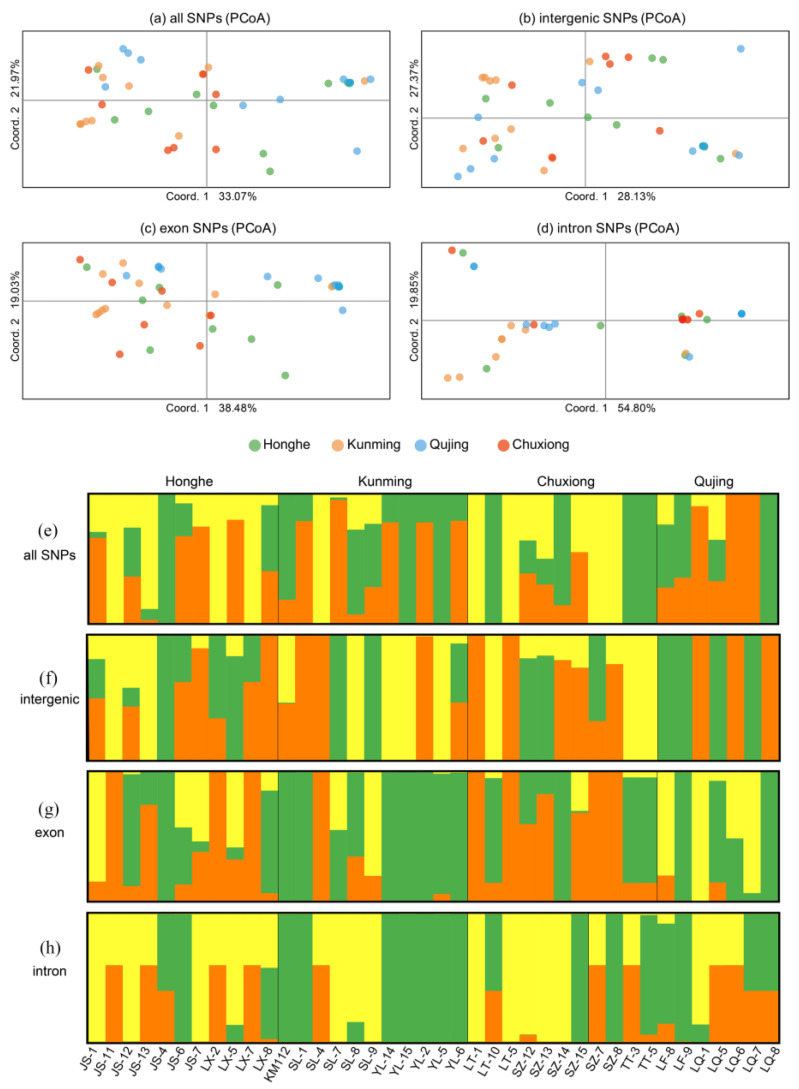
Genetic clusters and their distributions among four geographic regions obtained from the PCoA (**a**–**d**) and STRUCTURE (**e**–**h**) analyses of different datasets of mitochondrial genomes. Different colors represent different genetic clusters.

The principal coordinate analysis (PCoA) in Figure 6a–d revealed that all SNPs, intergenic SNPs, and exon SNPs in the samples from four different geographical populations could not be clearly separated. They were widely distributed, whereas the intron SNPs could clearly divide all samples into three genetic populations, of which PCoA axis 1 and axis 2 accounted for 54.80% and 19.85% of the genetic variation of the introns, respectively.

Figure 6e–h shows that in the STRUCTURE analysis, the best K values of the four types of SNPs were all 3, indicating that there were three genetic populations in all 40 samples. There was frequent gene exchange in the mitochondrial genome of *T. ganbajun* because the same geographical group was found to have all three genetic elements simultaneously. The exception was in the exon region, in which five samples from Yiliang (YL-14, YL-15, YL-2, YL-5, and YL-6) exhibited highly similar genetic structures, and four samples also had completely identical genetic structures in the intron region.

Among the total 24 pairs of genetic differentiation (Fst) values, the lowest Fst (0) was observed between Honghe (HH) and Chuxiong (CX) in the intron region, whereas the highest Fst = 0.275 occurred in the exon region between Chuxiong and Qujing. Ten pairs of natural populations showed statistically significant differentiation (*p* < 0.05). The Fst values of all SNPs ranged from 0.029 to 0.170; 2 out of 6 were significant, and one pair showed extreme significance. The range of Fst values in the exon region was relatively large (from 0.033 to 0.275), and more pairs showed significant genetic differentiation (3/6). Intron Fst values ranged from 0 to 0.157, whereas only one of the six pairs of genetic differentiation was significant (Table S4).

The overall level of all SNPs, both within and among the populations, contributed significantly to genetic differentiation (*p* = 0.007) (Table S5). Moreover, 10% of the genetic differentiation was between populations, and 90% of the genetic differentiation was from within geographic populations. Other results involving different gene regions in the mitochondrial genome of *T. ganbajun* indicated that in the intergenic region (*p* = 0.013) and the exon region (*p* = 0.002), genetic differentiation within and among populations contributed significantly to the overall genetic variation. In the exon region, 13% of the genetic variance was from among geographic populations, and 87% were from within populations. The gene flow indicated by the exon region was the smallest (Nm = 3.366), which is consistent with the results of its significant genetic differentiation. However, a different division level of populations indicated by the intron region did not contribute significantly to the overall genetic variation (*p* = 0.066). Thus, the gene flow in the intron region was the largest (Nm = 6.482) (Table S6).

## 3. Discussion

Bayesian clustering using the 13 concatenated core protein-coding genes conserved in the mitochondrial genome revealed that there was genetic differentiation within the species. By comparing the mitochondrial genomes of 40 samples, we found that the main differences in the standard protein-coding genes were not only manifested in the dynamic introns but also in the multicopy numbers of heterogeneous genes. In the present study, we found a new occurrence of heterogeneity in the *nad5* gene. In addition to the heterogeneous introns present in the *cox1* gene, as previously reported [30], we found multiple copies of the *cox1* and *nad5* genes. Because the length of the two heterogeneous genes in each sample accounted for 13.14–18.56% of the length of the mitochondrial genome, the existence of mitochondrial heterogeneity markedly affects the composition and structure of the mitochondrial genome.

In addition, there were differences in the number of introns contained in the mitochondria of the different *T. ganbajun* samples. There was obvious intron gain or loss of polymorphisms in the *cox1*, *nad5*, *cox3*, *nad1*, and *cox2* genes, which was a major factor causing changes in the size of the host genes. Several studies on fungi have reported similar cases. For example, the analysis of the mitochondrial genomes of 74 basidiomycete species from 15 orders revealed that there was a significant correlation between the number of introns and the size of the basidiomycete mitochondrial genome [31]. A recent comparative analysis of 35 fungal species revealed structural dynamics in their genomes, with size variation primarily associated with non-coding elements [27]; Similarly, intergenic regions are believed to be the primary contributors to genome size variation in the genus *Tricholoma* [8]. Although most of these studies are based on interspecies comparisons, introns and intergenic regions are also major factors that cause mtDNA differences in the intraspecies mitochondrial genome comparison of *Pleurotus pulmonarius* [32].

Besides the change in the number of introns, other factors were correlated with the variation and occurrence of heteroplasmy in the mitochondrial genomes of our samples. The number of multicopy genes of *cox1* was significantly correlated with the number of GIYs. There was also a significant correlation between the number and length of the introns in the mitochondrial genome and HEG size, and also with the number and size of LD1, which is caused by a larger number of HEGs (61.67%) and the presence of the LD1 homing enzyme among the three homing endonucleases. LD1 is the homing enzyme with the largest number (4–9) and the largest size (1222–2838 bp). Recent studies have shown that the GIY-YIG and LAGLIDADG families have an important role in shaping the genome structure of fungi and contribute to the variation within and between species. Determining whether there are common HEGs in the mitochondria of different strains of the same species is of great significance for revealing the stability of these HEGs at the species level [33].

Comparative analysis of the mitochondrial genomes revealed that the gene order in *T. ganbajun* was relatively conserved. The only exception was the insertion of one of the multicopy genes of *nad5* with a segmental repeat sequence into the *cox1* multicopy gene in LQ-8. This is consistent with reports indicating that the accumulation of repetitive sequences in the mitochondrial genome of fungi causes rearrangements of their mitochondrial genes [9]. The existence of mitochondrial heteroplasmy provides us with a deeper understanding of the structural composition of mitochondria, nucleo-cytoplasmic interactions, and the underlying genetic mechanism. In the SL3–6 sample, in which we initially found heteroplasmy, a truncated homing endonuclease aI3 was inserted into the *cox1* gene, followed by nonsense mutation accumulation. Thus, there are three abnormal stop codons in the coding region, suggesting that the abnormal aI3 is an inactivated copy in SL3–6, but it has not been lost during long-term evolution. Maintaining mitochondrial function often involves having multiple functional copies of key genes and associated elements [34]. Therefore, to maintain the normal function of mitochondria in *T. ganbajun*, there may be some functional copies of the *cox1* gene or other functional HEs in the mitochondrial genome of the sample and others in nature.

### 3.1. Variations of HTs

There are two basic types of mtDNA sequence polymorphisms: nucleotide substitutions caused by point mutations and length variation that can result from varying copy numbers of an oligonucleotide sequence or large-scale genomic duplications. The propagation of any new mutation requires, if only briefly, a transition stage in which multiple forms of the mitochondrial genome coexist within a single individual, a so-called heteroplasmic state. Mutation rates that alter the copy number play an important role in the maintenance of heteroplasmy and the maintenance of sequence identity among repeats [35].

The analysis reveals several key observations about mitochondrial introns and exons. Exons exhibit significantly higher variability than introns, with a greater number of SNPs detected in exons compared to introns. Additionally, the nucleotide diversity of multicopy genes like cox1 and nad5 is notably higher than that of the 13 conserved protein-coding genes. This suggests that these multicopy genes experience more variation and diversity. The lower polymorphism observed in introns is likely due to the presence of homing endonucleases, which are relatively conserved. However, despite the general conservation, some introns, particularly those near the root of the phylogenetic tree, show considerable variation, while others, such as LD1, are more stable and abundant. Analysis at the level of the mitochondrial genome revealed that both the *cox1* and *nad5* genes exhibited significant heterogeneity. After verifying the authenticity of the existence of multiple copies of genes by fluorescent quantitative PCR, we found that these different heterogeneous fragments were present in different samples. The distribution was not the same and had a large variation. We further confirmed that the frequency of heterogeneity fragments in different geographical groups in Yunnan Province was different. Heterogeneity may arise through different pathways and mutate during transmission. First, the parent may be heterogeneous, containing two or more different types. Because the replication and division of mitochondria are not synchronized with the indirect division of cells, heterogeneous mitochondrial DNA is randomly distributed to daughter cells. Second, mutations may occur during mtDNA replication or division [36]. The length of fungal mtDNA is not related to evolutionary history, indicating that its structure and content are prone to recent and rapid evolutionary changes [27]. Subsequently, each individual inevitably contains many mutated forms of mitochondrial heteroplasm inherited maternally. Finally, leakage of paternal mtDNA may be an important source of heterogeneity [37].

### 3.2. The Effect of Heterogeneity

As evidenced by the similar base content ratios of closely related species, which can, to some extent, maintain population stability, heterogeneity has a positive effect on the genetic diversity of populations and may enable populations to adapt to variable environmental conditions and alleviate population bottleneck effects. For example, in the basidiomycetous species genus *Phellinus noxius*, mitochondria are swapped during the mating process and during mycelial fusion, a mechanism that assists in the restructuring of mitochondrial genotypes and the establishment of heterogeneity [28]. The phenomena of heterogeneity transmission in certain fungal and animal species are explained by selective forces or by strict regulation during the replication and segregation of mitochondrial nuclei [28].

Currently, the selective pressures that maintain heteroplasmy and drive the reproduction of natural *T. ganbajun* populations remain unidentified. In the human pathogenic yeast *Cryptococcus neoformans*, high temperature and ultraviolet light exposure result in changes in mitochondrial inheritance from uniparental to biparental, as well as promote mitochondrial heterogeneity and recombination [11]. Mitochondrial genomes are typically asexual and vulnerable to Muller’s Rachet due to their uniparental inheritance, which can lead to the irreversible accumulation of harmful mutations [38]. Heteroplasmy is essential for mitochondrial recombination; the widespread presence of heterozygous forms of *cox1* and *nad5* in *T. ganbajun* populations suggests effective mechanisms that prevent Muller’s Rachet in their mitochondrial genomes. Mitochondrial diversity in different regions and geographic populations appears unrelated, indicating a unique evolutionary history for mitochondria in *T. ganbajun*. These genetic patterns likely result from background selection linked to highly deleterious mutations in the tightly linked mitochondrial genome. Therefore, under the special climate and geographical environment of Yunnan Province, different selection pressures may cause extensive and diverse heterogeneity of mitochondrial genes over a short period. High temperatures and intense UV radiation are two potential selection factors in *T. ganbajun* in Yunnan. These conditions promote mitochondrial heterogeneity and reorganization as an adaptive strategy, which lowers the rate at which harmful mutations can accumulate in the mitochondrial genome.

Heteroplasmy may be inherited from heterogeneous parents or can result from mating behavior (usually matrilineal in animals). We hypothesize that genetic variability in wild populations is likely to be inherited along ancestral mitochondria because of the extraordinary stability of heterogeneity among samples. Moreover, *T. ganbajun* in Yunnan may exhibit persistent and widely distributed genetic heterogeneity as an adaptive response to the regional environment. Therefore, these heterogeneous gene types may be used as molecular markers to study the genetic diversity of *T. ganbajun* and provide a method for the future study of intraspecific polymorphism and mitochondrial inheritance in *T. ganbajun*.

### 3.3. Intron Dynamics

The distribution patterns of introns in *T. ganbajun* were not consistent with their phylogenetic relationships. For example, the normal intron near the root of the phylogenetic tree was distributed in 10 different host genes, whereas the insertion sites were even more different; the insertion of introns seems to have occurred many independent times through mt genome evolution. Although introns showed considerable polymorphism, variations in insertion positions across different host genes and samples led to introns with only partial sequence similarity.

The *cox1* gene was the most common site for intron insertion in our species. It contained 187 of the 707 identified introns, which is consistent with the evidence from various fungal lineages confirming remarkably high density in intron content, particularly in *cox1* [39]. For example, in the mtDNA analysis of *Ophiocordyceps. sinensis*, *Hypocreales* had the highest number of introns (54 with 67.6% coverage), and *cox1* was the most abundant with 14 [40]. Previous studies have also shown that the cox1 gene is the most intron-containing and largest host gene of basidiomycetes. Therefore, *cox1* intron dynamics have a significant impact on the size of the mitochondrial genome of basidiomycetes [31].

Mitochondrial introns are considered selfish genetic elements, whose evolution and movement are complex with different levels and variations [33]. The results of the present study indicated that the same sample had different numbers but the same type of introns. There were gains and/or losses of whole introns as well as gains and/or losses of fragments of introns. It was hypothesized that introns are highly abundant in ancestral genes and that a general evolutionary process results in the selective loss of introns [41]. The intron gain/loss mechanism makes *cox1* of *Agaricus bisporus* the mitochondrial gene with the most group I introns [42]. The most commonly observed mechanism of intron loss is the replacement of an intron-containing gene following homologous recombination between an intronless cDNA (single-stranded DNA complementary to the mRNA) and the corresponding DNA [43].

The acquisition of introns may also occur through homing pathways. Intron homing refers to the movement of introns with HEGs to non-introns through various mechanisms, such as double-strand breaks, single-strand gaps, or homologous recombination [16]. Intron homing occurs at various levels and may be transferred between unassociated genes, alleles, or different chromosomes [44]. Most introns contain complete or truncated ORFs associated with HEs of the LAGLIDADG and GIY-YIG families [16]. These HEGs are thought to be responsible for the intron movement [45]. HEG variation is diverse and generates genetic diversity not only through homing events and radical changes in the mitochondrial local genome structure into which they insert but also through replication [19]. This concept was further supported in the present study, in which LD1 homing enzymes were characterized by the presence of two adjacent copies of the LD1 gene in the *atp6* and *cox1* genes. The *cox1* gene in the present study showed the most diverse introns with HEG variability. It contained four different intron types, and it was the gene with the most introns, with 135 out of 187 introns containing either complete or degenerative putative HEs.

Introns may also be acquired by HGT, in which introns can spread horizontally in different species and even kingdoms [46]. In the present study, homologous sequences of 10 homing enzymes were identified in 39 species in the NCBI database. The HGT of these HEs was supported by a high percentage of homology with other fungal species and even bacteria, which indicates that there are many complex transfer mechanisms. In the phylogenetic analysis of HGT HEs, the species of different genera were included in the same clade, and species of the same genus were dispersed into different clades, indicating that the genetic variation of HEs was diverse and not consistent with the phylogenetic relationship of species. Furthermore, the levels of HGT were also rich and varied. Intron transfer to other genomic regions with less sequence similarity can further spread under stress-induced conditions [20], which leads to more intron-rich transfer events. Studies have demonstrated that the number of insertion sites and introns varies between species, even among those that are closely related. This is consistent with the idea that intron loss and insertion occur periodically because of horizontal transfer and that fungal species may have higher rates of HGT. Numerous species, including *Aspergillus*, *Penicillium*, *Nectarineae*, and *A. chrysoporthe*, have had their horizontal intron transfer pathways in fungal mitochondrial DNA documented. There are reports of both group I and II introns being transferred horizontally from fungi and algae to various eukaryotes, including early-branching animals [47]. Furthermore, HGT is more common between species from different families than it is within the order *Hypodontia* alone [27]. In the mitochondrial genes of *Sclerotinia borealis*, nine introns were identified; however, only three showed a resemblance to the mitochondrial genes of the order *Helotiales* [27]. In vascular plants, group I introns most likely came from lateral transfers. Angiosperms experience a similar scenario when a splicing bacteria receives a second HGT event from a fungus that is comparable to *P. anserina* [48].

The three HEs detected in the present study were embedded in protein-coding genes with separate ORFs, whereas the normal intron had no ORF. HEG may be an independent ORF in an intron, or it may be integrated with an upstream exon [16]; thus, the former three homing endonucleases may belong to free HE. Free HEGs exist at the root of the phylogenetic tree, which may have determined the ancestors of free HEGs and the transition of HEGs themselves from independent mobility to intron-homing status [16]. This hypothesis is further supported by the results of the present study, in which the clades of normal intron and different HEGs were cross-distributed between the clades of different types of introns. This suggests that HEGs and normal introns co-evolved and that HEGs are undergoing a transition from independent mobility to intron-homing states. Previous studies indicate that HEGs were initially mobile elements independent of host introns, but they subsequently formed composite mobile elements by targeting the same sequences as introns. These composite introns jumped in a cis-acting fashion to other genes with similar target sequences, which resulted in homing [16]. Therefore, the co-evolution of introns and HEGs was expected, and HEGs became a necessary component of introns, which provides advantages for the survival of organisms that contain introns with HEGs [16]. Furthermore, it was discovered that ecological parameters including temperature, rainfall, latitude, and altitude may have an impact on intron dynamics [29]. Among the matsutake samples, distinct dynamic features were observed in three distinct geographic contexts. In addition, deletion of *cox1* P372, a locus-specific variation, was identified in this study. This variation may be used as a molecular marker to differentiate matsutake from other closely related species linked to mitochondrial inheritance [49].

### 3.4. Population Structure

In *T. ganbajun*, mitochondrial gene flow is common, but there is some genetic differentiation among geographic populations. Gene flow varies across different regions of the genome, with introns showing more gene flow than intergenic regions, and intergenic regions showing more gene flow than exons. Exons have higher Fst values than introns, indicating greater genetic differentiation. For example, the intron region in Yiliang’s five samples was identical, while exon differentiation was limited. PCA results support this, showing that intron SNPs can be divided into three distinct genetic structures, while exons cannot. Additionally, nucleotide polymorphism varies by region and population: exons show the highest variation in Honghe and Chuxiong, while introns show the highest variation in Kunming and Qujing.

The evolutionary rates of intron and exon sequences are not the same [50], and the imbalanced composition of introns and exons in the genome increases the susceptibility of introns to selection pressure and other natural selection processes [51]; Furthermore, introns have their own non-coding RNAs that are independent of host gene function. This suggests that introns are not just “fillers” in the genome but also have important roles in the cell, such as controlling protein synthesis and gene expression. This results in a more distinct genetic differentiation and a faster evolutionary pace of introns compared with other parts of the genome [52]. We showed that considerably more distinct genetic clusters with intron SNPs were observed. In a study of the mitochondrial genome of *Cordyceps militaris*, the levels of variation in exons and introns of protein-coding genes were similar, whereas incomplete exon amplification resulted in higher intron variation in the RNL gene compared with that in exons [50]. In the present study, all types of SNPs were scanned from the entire mitochondrial genome, and there was no incomplete gene in any region. This may be due to the existence of some obvious structural variation in the mitochondrial genome of our species, and further analysis of mitochondrial genome structural variation is needed.

Geographic distance, historical differentiation, anthropogenic influences, and environmental changes brought on by climate change can all affect the demographic structure of *T. ganbajun*. As previously mentioned, wind is one example of a natural force that facilitates gene flow between distant nations. Spores can travel great distances when the weather is windy and dry [9]. Wind-borne propagation encourages genetic recombination and adds to the general genetic similarity of species in certain areas. A physical barrier formed in different ecosystems, when paired with possibly distinct selective pressures and/or genetic drift, can result in variations in the frequency of specific alleles and allele combinations, thus preserving genetic diversity and improving adaptation to local environments.

## 4. Materials and Methods

### 4.1. Sampling, DNA Isolation, and Genome Sequencing

The fruiting bodies of *T. ganbajun* were collected from Yunnan Province, southwest China. All 40 samples with heterogeneity in the *cox1* gene were selected from our previous study [30]. Heteroplasmy was characterized by the presence of two types of introns residing at adjacent but different sites in the *cox1* gene within an individual strain [28]. Details of the samples are shown in Table 2. Construction of sequencing libraries and sequencing of all 40 samples were performed using an Illumina HiSeq 2500 Platform by Zhihuike Co., Ltd. (Wuhan, China). The fastp program and fastQC software Version 0.11.9 were used to assess the quality of the original sequencing data to obtain clean reads.

For population genetic analysis at the mitochondrial genome level, the 40 samples were divided into four geographic populations based on their prefecture affiliations (an administrative level of government between the county and the provincial governments), namely Honghe (HH), Kunming (KM), Qujing (QJ), and Chuxiong (CX).

### 4.2. De Novo Assembly, Annotation, and Comparative Analysis of Mitogenomes

The assembly and annotation for all mitogenomes analyzed in this study followed the methods described previously [53]. Briefly, all mitogenome reads were extracted from whole genome sequencing data with the published *T. ganbajun* mitogenome (GenBank accession number KY245891) as a reference. They were used to identify homology with our mitogenome sequence reads using the NCBI BLAST algorithm. Next, we assembled the sequencing data using SPADES [54] and SOAPdenovo2 software [55], and the preliminary assembly was compared with the NT database. The sequences annotated as mitochondrial genomes and loops were screened by comparing the mitochondrial genomes of closely related species using MUMmer 3.0 [56]. High-quality complete mitogenome maps were obtained.

Gene structure prediction of fungal genomes was performed using Glimmer v3.02 software [57], which used Interpolated Markov Models to identify coding regions and non-coding genes with low false-positive prediction results. tRNA prediction of fungal mitochondrial genomes was performed using tRNAscan-SE v1.3.1 [58], and rRNA was predicted using RNAmmer v1.2 [59]. Furthermore, non-coding RNA (ncRNA) structures were identified by Rfam v12.0 [60], and intron structures were predicted using AUGUSTUS web server (http://augustus.gobics.de/submission).

We compared mitogenomes from all 40 samples of *T. ganbajun* in terms of size, GC content, base composition, gene and intron numbers, and gene arrangement to assess conservation and variation between the species’ mitogenomes. The corrplot package in R was used to conduct a correlation analysis of various factors [61], then the gggenes package was used to determine the composition and arrangement of the gene structures [62], as well as the positioning of the introns and HEs. Finally, the ggplot2 package was used for visual plotting [63].

### 4.3. Existence of Multicopy Heterogeneity in the nad5 and cox1 Genes Verified by qPCR

All 40 *T. ganbajun* samples contained multiple copies with the two genes, *nad5* and *cox1*, and different copies comprised different heterogeneous types (HTs). In case of errors in sequencing or assembly procedures, further verification of the authenticity of the existence of these multicopy HTs is required. Eight samples with all 28 multicopy HTs represented by each specimen were verified by fluorescent quantitative PCR based on our previously described method [28]. The details of the samples and primer sequences for amplifying HTs are listed in Tables S1 and S2. The nuclear single-copy gene β-tubulin was used as a reference and the relative copy numbers of each type in the *nad5* and *cox1* genes within each sample were calculated by the 2^−∆∆Ct^ algorithm [64].

### 4.4. Population Genetics

We obtained four SNP datasets from the mitogenome of *T. ganbajun*. The analysis of all SNPs, intergenic SNPs, exon SNPs, and intron SNPs and the comparative analysis of population genetics using these SNP datasets were performed separately. DNA polymorphisms were calculated for each population and each region using DnaSP v6.10.01 software (Universitat de Barcelona, Catalonia, Spain) [24], including nucleotide diversity (Pi), parsimony information sites, and singleton variable sites.

A Nei’s pairwise genetic distance matrix was generated based on the populations using GenAlEx 6.5 software (The Australian National University, Canberra, Australia) [65]. The Mantel test in GenAlEx was used to detect correlations between the levels of Nei’s pairwise genetic distances and geographical distances among populations [66]. In addition, the same software was used to perform an analysis of molecular variance to estimate the relative contributions of geographic separation to the overall genetic variation. The program STRUCTURE v2.3.3 (Stanford University, Stanford, CA, USA) [67] was used to estimate the number of genetic clusters.

## 5. Conclusions

In this study, a wide range of heterogeneity, polymorphisms, and structural variations was revealed by mitochondrial genome sequencing and annotation of 40 *T. ganbajun* samples. The presence of heterogeneous genes, introns, and HEGs was found to greatly affect the composition and evolution of mitochondrial genes. This enhances our understanding of mitochondrial genome structure, population dynamics, and the evolution of *T. ganbajun*. Thus, this study is of great significance for resource conservation and the genetic diversity of *T. ganbajun* and provides a strong basis for further research on the evolution and effects of mitochondrial heterogeneity in eukaryotes. Mitochondrial inheritance is related to the structural variation of mating-type genes in fungi. An in-depth analysis of nucleoplasmic interactions should be performed at the mitochondrial genome and nuclear genome levels in the future. Furthermore, multicopy heterogeneous fragments have the potential to be used as molecular markers for the identification and genetic diversity analysis of *T. ganbajun* and other edible mushrooms in Yunnan.

## Figures and Tables

**Table 1:** Characterization of the 40 mitochondrial genomes of *T. ganbajun*.

Strains	Genome Size (bp)	GC Content (%)	Standard Protein-Encoding Genes (PCGs) Size (bp)	Tot. Multicopy Genes Size (bp)	HEGs Size (bp)	Normalintrons Size (bp) (No.)	No. HE	Tot. Introns	GenBank Accession
JS-1	65,668	26.04	22,096	11,172	4022	1567 (5)	11	16	PQ126529
JS-11	65,543	25.90	24,854	12,013	4022	1397 (6)	11	17	PQ149496
JS-12	64,773	25.89	22,177	11,135	4121	1850 (8)	11	19	PQ149497
JS-13	66,588	26.04	24,816	11,975	4723	2078 (8)	13	21	PQ149498
JS-4	64,130	25.85	22,505	8428	3456	2101 (6)	9	15	PQ149493
JS-6	65,686	25.86	23,799	11,968	3150	1500 (7)	9	16	PQ149494
JS-7	68,725	25.94	24,078	10,039	4116	2396 (8)	12	20	PQ149495
KM112	64,682	25.86	21,813	9868	4121	1940 (6)	11	18	PQ149499
LF-8	65,622	26.00	23,710	10,746	4022	1129 (4)	11	15	PQ149500
LF-9	64,911	25.88	23,941	10,813	3162	2497 (9)	9	18	PQ149501
LQ-1	67,472	25.93	25,158	12,040	4425	1980 (6)	12	18	PQ149502
LQ-5	65,082	25.87	20,697	9655	4431	1157 (6)	12	18	PQ149503
LQ-6	64,913	25.93	23,545	9925	4121	2286 (7)	11	18	PQ149504
LQ-7	68,859	25.95	25,190	11,679	4022	2396 (8)	11	19	PQ149505
LQ-8	67,071	25.76	23,873	10,000	3107	2085 (7)	8	15	PQ149506
LT-1	65,543	25.89	24,854	12,013	4022	1397 (6)	11	17	PQ149507
LT-10	65,796	26.03	23,723	9874	4121	2343 (8)	11	19	PQ149509
LT-5	66,733	26.03	24,854	12,013	4376	1397 (6)	11	17	PQ149508
LX-2	65,573	26.03	25,012	12,171	4022	1408 (6)	11	17	PQ149510
LX-5	65,635	25.98	24,381	11,127	4061	2430 (9)	11	20	PQ149511
LX-7	65,709	26.05	24,952	12,111	4022	1300 (5)	11	16	PQ149512
LX-8	64,567	26.23	19,515	9205	3580	1164 (6)	11	17	PQ149513
SL-1	64,806	25.86	23,091	9868	4121	2383 (8)	11	19	PQ149514
SL-4	64,475	26.11	22,798	10,746	4332	1206 (5)	12	17	PQ149515
SL-7	67,542	25.92	22,654	10,585	4949	2059 (8)	14	22	PQ149516
SL-8	65,207	25.83	21,530	8998	3249	1806 (6)	9	15	PQ149517
SL-9	65,631	26.00	23,710	10,746	4022	1129 (4)	11	15	PQ149518
SZ-12	65,060	25.93	25,335	11,461	4407	1941 (6)	12	18	PQ149521
SZ-13	66,778	26.07	24,842	10,746	4236	1760 (7)	12	19	PQ149522
SZ-14	67,314	25.99	20,216	9322	4071	2688 (8)	12	20	PQ149523
SZ-15	64,879	25.95	22,862	9356	3800	2134 (7)	11	18	PQ149524
SZ-7	65,570	26.03	24,854	12,013	4332	1397 (6)	12	18	PQ149519
SZ-8	65,539	26.04	24,854	12,013	4022	1397 (6)	11	17	PQ149520
TT-3	65,809	25.90	23,779	9874	4121	2351 (7)	11	18	PQ149525
TT-5	65,824	25.90	25,053	11,148	4121	2733 (9)	11	20	PQ149526
YL-14	64,831	25.86	23,091	9868	3586	2428 (9)	10	19	PQ149530
YL-15	64,414	25.69	20,413	8468	3456	1263 (5)	9	14	PQ149531
YL-2	64,840	25.85	22,642	11,069	3237	1729 (8)	9	17	PQ149527
YL-5	66,855	25.80	24,501	10,707	3772	2217 (7)	10	17	PQ149528
YL-6	64,799	25.87	21,441	9868	4121	1612 (7)	11	18	PQ149529

**Table 2:** Geographical origin and distribution of 40 *T. ganbajun* samples.

Geographic Population	Sampling Site	Long. (E)	Lat. (N)	Region Sample Size	Local Geographic Population Sample Size
Honghe (HH)	Luxi	103.76	24.52	11	4
	Jianshui	102.79	23.64		7
Kunming (KM)	Kunming	102.83	24.88	11	1
	Yiliang	103.08	24.55		5
	Shilin	103.2	24.45		5
Qujing (QJ)	Longtan	104.01	26.41	11	3
	Tangtang	104.21	26.48		2
	Shizong	103.59	24.49		6
Chuxiong (CX)	Lufeng	102.04	25.09	7	2
	Luquan	102.28	25.33		5

**Table 3:** Polymorphisms of SNPs in different datasets of the mitochondrial genomes of *T. ganbajun*.

SNPs Type	All Sites	Parsimony Information Sites	Singleton Variable Sites	Nucleotide Diversity (Pi)	Pi of per Population			
					Honghe	Kunming	Qujing	Chuxiong
All SNPs	2463	1911	552	0.230	0.240	0.206	0.222	0.246
Intergenic SNPs	1467	1160	310	0.233	0.215	0.199	0.235	0.240
Exon SNPs	921	692	229	0.263	0.282	0.215	0.199	0.259
Intron SNPs	66	53	13	0.252	0.227	0.254	0.258	0.199

## Data Availability

The raw data supporting the conclusions of this article will be made available by the authors on request.

## Supplementary Materials

The following supporting information can be downloaded at https://www.mdpi.com/article/10.3390/ijms25169013/s1.
