# Supplementary material for: Exploring Mitochondrial Heterogeneity and Evolutionary Dynamics in Thelephora ganbajun through Population Genomics
Source: Int J Mol Sci. 2024 Aug 19;25(16):9013. doi: 10.3390/ijms25169013 (PMC11354633; doi:10.3390/ijms25169013)
Supplement: Supplementary file 1 [file ijms-25-09013-s001.zip › ijms-3118998-supplementary/Table S2 Genetic clusters.pdf]

Table S2: Distributions of 28 multi-copy genes tested in this study.

| | | JS-7 | LQ-1 | LQ-8 | LX-5 | SL-7 | SZ-14 | SZ-8 | TT-5 |
|---|---|---|---|---|---|---|---|---|---|
| reference | TUB | 1 | 1 | 1 | 1 | 1 | 1 | 1 | 1 |
| cox1 1 | Cmc1-1 | | | | | | | 1 | |
| | Cmc1-2 | 1 | | | 1 | | | | 1 |
| | Cmc1-3 | | | | | 1 | 1 | | |
| | Cmc1-4 | | 1 | | | 1 | | | |
| cox1 2 | Cmc2-1 | | | | | | | 1 | |
| | Cmc2-2 | | | 1 | 1 | | | | |
| cox1 3 | Cmc3 | 1 | 1 | 1 | 1 | 1 | 1 | 1 | 1 |
| cox1 4 | Cmc4-1 | | 1 | | | | | 1 | |
| | Cmc4-2 | 1 | | | | | | | |
| cox1 5 | Cmc5-1 | 1 | | | | | | 1 | |
| | Cmc5-2 | | | 1 | 1 | 1 | 1 | | 1 |
| nad5 1 | Nmc1-1 | 1 | 1 | 1 | | | 1 | 1 | 1 |
| | Nmc1-2 | | | | 1 | | | | |
| | Nmc1-3 | | | | | 1 | | | |
| nad5 2 | Nmc2-1 | 1 | 1 | 1 | 1 | 1 | 1 | 1 | 1 |
| | Nmc2-2 | | | 1 | | | | | |
| nad5 3 | Nmc3 | 1 | 1 | 1 | 1 | 1 | 1 | 1 | 1 |
| nad5 4 | Nmc4-1 | | | 1 | | 1 | | 1 | 1 |
| | Nmc4-2 | 1 | | | | | | | |
| | Nmc4-3 | | | | | | 1 | | |
| | Nmc4-4 | | 1 | | 1 | | | | |
| nad5 5 | Nmc5-1 | | | | | 1 | | 1 | 1 |
| | Nmc5-2 | | | 1 | | | | | |
| nad5 6 | Nmc6 | 1 | 1 | 1 | 1 | 1 | 1 | 1 | 1 |
| nad5 7 | Nmc7-1 | 1 | 1 | 1 | 1 | 1 | 1 | | |
| | Nmc7-2 | | | | | | | 1 | |

| | |
|---|---|
| Nmc7-3 | 1 |
