# Supplementary material for: Exploring Mitochondrial Heterogeneity and Evolutionary Dynamics in Thelephora ganbajun through Population Genomics
Source: Int J Mol Sci. 2024 Aug 19;25(16):9013. doi: 10.3390/ijms25169013 (PMC11354633; doi:10.3390/ijms25169013)
Supplement: Supplementary file 1 [file ijms-25-09013-s001.zip › ijms-3118998-supplementary/Table S3 Primers.pdf]

Table S3: Primers for amplification of multi-copy fragments from *cox1* and *nad5*

Gene	Forward sequence (5'-3')	Reverse sequence (5'-3')
TUB	TCTTTCTGCCCTCCATTCAA	TCCTTTCCAACGGTGTAGTGA
Cmc1-1	ATGAACTTGCCCTATTATTTTAATAA	AAAGTTATAGCTATAGAAGGTGAAGTG
Cmc1-2	CTAATTTAAGACAGTCTACACACAATGTA	TGATTATTATCAAGCATTAAATTATAAAC
Cmc1-3	ATTCACTTAGGAGCACCAGATTAC	TCTTGTGAATAGAGAGGCAAAGA
Cmc1-4	TATTCACAAGAAAGTGGTGATAATAC	GAAACTTAGAACACCTTCGAAGA
Cmc2-1	CGATTTAACCGATGATGAAATA	TTATATAATTATGTAACCAATTGCTTC
Cmc2-2	AAGCTGATCTACCTTTAGCTAATTA	GAGGTTATGTTTTATGGCAAATA
Cmc3	TACTTGGGAACAGGTTCTTGA	AAATAAGCGATTTGGTTGTAGT
Cmc4-1	TCAGGATTCTATTATTGGACTCCA	ATAGTAAATCCAAATCTGAATTATTCC
Cmc4-2	ATCATTTTGGTTATTACCACCATC	GAATGAGAAACAATACTAGCTAATGG
Cmc5-1	CTATCTTATTTGGTTATATTATTTATGACC	ATCACCATTAAATAGTACAGATGAAGTA
Cmc5-2	GGAATGCCTAGAAGAATCCCTG	TCTTTACCATTAGCGAATAAGTCATA
Nmc1-1	CAGTTATTGTTGGAGGTGTATTTAC	TAGCTGTTTGTATTCCAGCATC
Nmc1-2	CACCTAATAATTCTAATTTAGAAGAATTA	CATTATTCATATCATAAACTCTATTTGC
Nmc1-3	CACCTAATAATTCTAATTTAGAAGAATTA	TTATTCATATCATAAACTCTAGTTTCTAC
Nmc2-1	GCTGGTATGATTGTAACAGCTGTTA	CATTTGAATCCAATTTAGGTAAAGTG
Nmc2-2	CCTACTAATTATGTAGATGCCGAAGT	GGGTTAGTCGCTGAACTTCCTA
Nmc3	TCAATATCAGAATTACCTGAAACTGTT	CCATAATGATGATGTAGGTGTTTGT
Nmc4-1	GCAGCAATATTAGCATTCACA	CTCCCATTAATAATAATAAACCTATG
Nmc4-2	GATATAAATTATTTATCTATTTTACCTC	AAACATCCATAGTCATAGAATATTTT
Nmc4-3	TTTATTGATACTATGGATTATGATATAAT	TAAATTTAGAAAGACTAATAGAACCATC
Nmc4-4	TTTCATTAGCTTATTGGATAATGGA	TACTGAAAGATTCGGTACAAAGAAG
Nmc5-1	GTGATGGTTCTATTAGTCTTTCTAAT	AGGTAATAAAATATTCGGATATGC
Nmc5-2	CAGTTATTCTTTGTACTGAATCTTTCAC	AAAAATCTTTATGCATATACGGTTTG
Nmc6	ATGGGTGTGTTTAATACTTTAAGAGG	TTGTTTAATTCTTGATATTTAGTTTCATG
Nmc7-1	ATAATGGTGTAATTACTACTTACGCTC	GAATTTTTATTCTGTGGAGATAAAAC
Nmc7-2	ATAATGGTGTAATTACTACTTACGCTC	AATAAAATTAATTGTCCAATAAATTG

Nmc7-3	GTAAAATATATCTATTCTACTTTAAAGGC	ATTGAAAATGTAGTATTTAATGGAGT

Notes: Names of fragments start with C are the multi-copy genes of *cox1*, and those start with N are from *nad5*.
