# Supplementary material for: Exploring Mitochondrial Heterogeneity and Evolutionary Dynamics in Thelephora ganbajun through Population Genomics
Source: Int J Mol Sci. 2024 Aug 19;25(16):9013. doi: 10.3390/ijms25169013 (PMC11354633; doi:10.3390/ijms25169013)
Supplement: Supplementary file 1 [file ijms-25-09013-s001.zip › ijms-3118998-supplementary/Table S5 Pairwise Fst value of different datasets in mitochondrial genomes.pdf]

Table S5: Pairwise Fst value of different datasets in mitochondrial genomes

	Pops	HH	KM	QJ
All SNPs	KM	0.103*		
	QJ	0.029	0.149*	
	CX	0.065	0.048	0.170**
Intergenic	KM	0.108*		
SNPs	QJ	0.014	0.102*	
	CX	0.052	0.053	0.101*
Exon SNPs	KM	0.093*		
	QJ	0.052	0.222**	
	CX	0.087	0.033	0.275**
Intron SNPs	KM	0.157*		
	QJ	0.005	0.077	
	CX	0	0.125	0.028

Note: $p<0.05$, significant, *; $p<0.01$, extremely significant, **.
