# Supplementary material for: Exploring Mitochondrial Heterogeneity and Evolutionary Dynamics in Thelephora ganbajun through Population Genomics
Source: Int J Mol Sci. 2024 Aug 19;25(16):9013. doi: 10.3390/ijms25169013 (PMC11354633; doi:10.3390/ijms25169013)
Supplement: Supplementary file 1 [file ijms-25-09013-s001.zip › ijms-3118998-supplementary/Table S6 AMOVA analysis.pdf]

Table S6: AMOVA analysis of mitochondrial genomes

	Source	df	SS	MS	Est. Var.	%	Stat	Value	P	Nm
all	Among Pops	3	1716.423	572.141	29.570	10%	PhiPR			4.716
SNPs	Within Pops	36	10048.727	279.131	279.131	90%	PhiPT	0.096	0.007	
	Total	39	11765.150		308.728	100%				
Interg	Among Pops	3	867.962	289.321	12.820	7%	PhiPR			6.334
enic	Within Pops	36	5946.338	162.398	162.398	93%	PhiPT	0.037	0.013	
SNPs	Total	39	6714.300		175.219	100%				
Exon	Among Pops	3	806.832	268.944	16.170	13%	PhiPR			3.366
SNPs	Within Pops	36	3919.143	108.865	108.865	87%	PhiPT	0.129	0.002	
	Total	39	4725.975		125.035	100%				
Intron	Among Pops	3	41.628	13.876	0.607	7%	PhiPR			6.482
SNPs	Within Pops	36	283.247	7.868	7.868	93%	PhiPT	0.072	0.066	
	Total	39	324.875		8.475	100%				
